# Helping patients to reduce tobacco consumption in oncology: a narrative review

**DOI:** 10.1186/s40064-016-2798-9

**Published:** 2016-07-20

**Authors:** Claudio Lucchiari, Marianna Masiero, Andrea Botturi, Gabriella Pravettoni

**Affiliations:** Department of Philosophy, Università degli Studi di Milano, Milan, Italy; Department of Oncology and Hemato-Oncology, Università degli Studi di Milano, Milan, Italy; Applied Research Unit for Cognitive and Psychological Science, European Institute of Oncology, Milan, Italy; Department of Neurooncology, Fondazione IRCCS Istituto Neurologico C. Besta, Milan, Italy

**Keywords:** Oncology, Cigarette smoking, Smoking cessation, Decision-making

## Abstract

The present overview focuses on evidence of smoking cessation approaches in oncology settings with the aim to provide health personnel a critical perspective on how to help their patients. This narrative review is structured in two main sections: the first one describes the psycho-cognitive variables involved in the decision to continue smoking after a cancer diagnosis and during the treatment; the second section relates methods and tools may be recommended, being evidence-based, to support smoking cessation in oncology settings. Active smoking increases not only susceptibility to common cancers in the general population, but also increases disease severity and comorbidities in cancer patients. Nowadays, scientific evidence has identified many strategies to give up smoking, but a lack of knowledge exists for treatment of nicotine dependence in the cancer population. Health personnel is often ambiguous when approaching the problem, while their contribution is essential in guiding patients towards healthier choices. We argue that smoking treatments for cancer patients deserve more attention and that clinical features, individual characteristics and needs of the patient should be assessed in order to increase the attempts success rate. Health personnel that daily work and interact with cancer patients and their caregivers have a fundamental role in the promotion of the health changing. For this reason, it is important that they have adequate knowledge and resources in order to support cancer patients to stop tobacco cigarette smoking and promoting and healthier lifestyle.

## Background

Tobacco cigarette smoking is the leading cause of preventable death in the world. In 2011 the World Health Organization (WHO) declared that smoking caused six million deaths annually, and in 2030 it will kill eight million people each year (WHO 2011).

Bio-physiological evidence suggests that smoking is the main cause of respiratory diseases (e.g. chronic obstructive pulmonary disease and pulmonary emphysema), cardiovascular events (e.g. aneurysm and stroke) and many cancers (esophagus, larynx, oral cavity, bladder, stomach, kidney, squamous cell carcinoma of the cervix; Ehlers and Bronars 2012). Statistical figures confirm that tobacco dependence causes 30 % of all cancers and 80 % of deaths from neck, head, and lung cancer (Morgan et al. 2011). Particularly, about 90 % of lung cancer is assignable to tobacco cigarette smoking (Nayan et al. 2011).

Despite this evidence, many patients still smoke regularly after a diagnosis of cancer. In 2013 the American National Cancer Institute published a scientific statement on treating tobacco dependence in the cancer population. The report highlighted that 50 % of individuals with cancer who smoke before diagnosis continue smoking during treatment, and that, for patients who gave up smoking, the relapse rate was very high (Toll et al. 2013). Data showed that 23–35 % of head and neck cancer patients, and 13–20 % of lung cancer patients who were smoking before their diagnosis, continued to smoke after the diagnosis and during treatment itself (Nayan et al. 2011), while de Bruin-Visser et al. (2012) reported that 35–72 % of head and neck cancer patients’ smoke despite the diagnosis.

Also women with breast cancer are reported as continuing to smoke after the diagnosis (Land et al. 2011). In this study, cigarette smoking was found to be negatively correlated to treatment adherence.

Continuing to smoke after a cancer diagnosis may produce several adverse health outcomes (Cropley et al. 2008). Epidemiological research shows that tobacco cigarette smoking decreases survival time (Gritz and Demark-Wahnefried 2009), it increases the risk of second primary malignancies (Zevallos et al. 2009) and the likelihood of recurrences (Schnoll et al. 2004). For instance, smokers with Hodgkin’s disease have an increased risk of a second primary lung cancer (McBride and Ostroff 2003) when compared to never-smokers under the same conditions. Also, it has been shown that tobacco use decreases survival rate after hematopoietic stem cell transplantation (Marks et al. 2009; Ehlers et al. 2011). Furthermore, cigarette smoking decreases treatment efficacy (Waller et al. 2010) and increases complications and side-effects (Schnoll et al. 2004) for many types of cancer. In this regard, clinical studies revealed that in head and neck (Blalock et al. 2011), and lung (Zhang et al. 2009) cancer patients, smoking behavior decreases the efficacy of radiotherapy, while increasing drug toxicity and side-effects (such as impaired pulmonary function, nausea, and dry mouth) (Nayan et al. 2011). Daniel et al. (2009) showed that patients with lung cancer who did not discontinue smoking reported more pain during the disease progression than former smokers and non-smokers. Similar concerns have also been observed in patients with bladder and renal cancer (Videtic et al. 2003). Finally, smoker patients have a high risk of developing postoperative complications than do non-smoker patients, for example showing poor wound healing (Thomsen et al. 2010a, b; see Table 1).

**Table 1:** Summary of the benefits related to smoking cessation during and after cancer treatment

Benefits of smoking cessation post-diagnosis
Physical dimension
Decreasing the side-effects of the therapy
Increasing treatment efficacy
Overcoming chemo resistance
Improving outcome of the surgery
Counteracting tumor progression
Reducing fatigue
Increasing activity level
Reducing the likelihood of developing second primary malignancies
Avoiding recurrence
Improving survival rate
Psychological dimension
Improving cognitive process
Enhancing psychological well-being
Increasing self-esteem
Reducing blame (when cancer is smoking-related)
Supporting self-efficacy health behavior-related
Improving mood
Health behaviors
Supporting healthy lifestyle
Promoting preventive behavior
Enhancing general quality of life of the patients (e.g. quality of sleep)

In spite of the evidence, there is still a significant lack of smoking treatment research for oncology patients. A cancer diagnosis may provide a useful teachable moment (Gritz 1991) for quitting smoking, but this chance is frequently undervalued by the healthcare system (Weaver et al. 2012).

A number of studies reported that oncology patients actually try to give up, but they generally make their attempts without help. For example, a recent study by Vaidya et al. (2014) reported that many patients keep on smoking even after a diagnosis of lung cancer. Khullar et al. (2013) stated that patients do not receive enough support from physicians. Indeed, 50 % of physicians do not regularly talk about antismoking strategies with patients. Cooley et al. (2009) have shown that despite the a broad diffusion of information about the benefits of smoking cessation after a lung cancer diagnosis, few patients actually succeed in their attempt and find little support from health personnel pre- and post-surgery. Confirming this evidence, a large online survey conducted by Warren et al. (2013) in 1507 members of the International Association for the Study of Lung Cancer revealed that though most physicians believes smoking cessation to be an important issues for their patients, only 40 % discuss quit options and only 39 % actively support patients.

Generally, oncologists provide wide-ranging suggestions regarding smoking-related risks, but they do not give detailed information regarding available treatments, nor do they offer a support program during the whole therapeutic journey (Weaver et al. 2012). This view has been recently supported by a qualitative study conducted by Champassak et al. (2014), which showed, through a series of interview, the mismatch between the physicians’ believe of the importance of smoking cessation programs and the actual behavior during visits. Indeed, physicians report a lack of confidence in their smoking cessation counseling ability. Furthermore, physicians rarely reported to use recommended strategies, even when they are acknowledged. Another particular case is that of hematologic cancers. Indeed, in a hematological oncology department for patients undergoing hematopoietic stem cell transplantation, quitting is a priority, since studies exist (Chang et al. 2004) associating smoking with worse outcomes. Despite this, Ehlers et al. (2011) showed that many hematologic patients continue to smoke after diagnosis. Also, authors noted the lack of studies addressing the issue of helping patients quit smoking (Ehlers and Bronars 2012), and the training of healthcare professionals in the management of the smoker patient.

Starting from this theoretical background, the main aim of this brief review is to sketch an overview of the available options to approach smoking dependence in an oncology setting. In order to achieve this goal, the paper has been structured into three conceptual sections. The first section describes the role of the psycho-cognitive processes that reinforce cigarette smoking behavior in patients with cancer. The second section discusses the cognitive-behavioral interventions and smoking cessation aids, focusing both on advantages and disadvantages, in in-patient and out-patient settings. Finally, we offer some suggestions for health professionals to overcome roadblocks and to improve the smoking cessation success rate in cancer patients.

## Method

This narrative review is structured in two main sections: the first one describes the psycho-cognitive variables involved in the decision to continue smoking after a cancer diagnosis and during the treatment; the second section relates methods and tools may be recommended, being evidence-based, to support smoking cessation in oncology settings.

Searches were conducted through MEDLINE, EMBASE, PubMed, SCOPUS, PsycINFO and Google Scholar. The following keywords or combinations were used: cigarettes smoking, cancer, oncology patient, oncology setting, smoking cessation, antismoking treatment, nicotine dependence, nicotine replace treatment, varenicline, bupropion, electronic cigarettes, smoking cessation counseling.

In the first section, we considered papers from 1990 to May 2016, using previous systematic reviews, national and international reports, and empirical studies. While in the second section, we included only controlled trials studies from 1990 to May 2016.

Articles in languages other than English and letters and editorials were excluded. The initial search identified 763 articles. An initial review of the titles abstracts of these articles by 2 of the authors identified 133 articles that were potentially relevant to the current review. The abstracts of these articles were then evaluated against the inclusion criteria by the authors, resulting in 35 articles being identified as eligible for inclusion, with an additional 6 articles identified during manuscript preparation, for a total of 41 articles. This review provides a narrative synthesis of the findings from previous key reviews and empirical studies identified in the literature search.

- Inclusion and exclusion criteria section 1:

We included article assessing the behavioral, psychological and cognitive characteristic of adult smokers after a diagnosis of cancer, including nicotine dependence level and motivation to quit. We also reviewed paper (review or empirical studies) that evaluated the rate of patients that continue smoking after a cancer diagnosis. We excluded editorial, opinion papers, letters and articles related to adult smokers after childhood cancer.

- Inclusion and exclusion criteria for section 2:

We included prospective controlled studies about adults patients after a diagnosis of cancer during their clinical treatment, measuring the effectiveness of a smoking cessation treatment on smokers who received a diagnosis of cancer and describing the smoking cessation methods used, including the type of provider and the clinical setting. We considered studies that specify the cancer sites of patients and stage. We excluded studies on mixed population (cancer patients and other diseases), cohort studies as well as surveys.

### Cognitive profile of the cancer patient who smokes

Westmaas et al. (2015), in a large prospective study on a US cohort (including more than 12,000 smokers) showed that a cancer diagnosis is associated with significantly higher rates of quitting smoking within both 2 and 4 years of the diagnosis. Almost one in three smokers who received a cancer diagnosis in their study had quit smoking within 2 years, compared with fewer than one in five smokers who had never received a cancer diagnosis. Moreover, the greater quitting rate among cancer-diagnosed smokers is maintained at 4 years. Interestingly, also non-smoking related diagnosis affected patients’ smoking habits. However, even though a diagnosis of cancer seem to be a true teachable moment, not all patients actually change their behavior. In particular, patients with preexisting chronic conditions that are linked to smoking (e.g. chronic obstructive pulmonary disease) have low rates of quitting after cancer diagnosis. Coherently, Burris et al. (2015) aggregating data across 131 studies on head and neck cancer patients, found that about 50 % of smokers patients continue smoking after diagnosis, while previous studies reported a range of 35–72 % (Ostroff et al. 2013).

These data may help in describing the typical cancer patient who continue smoking after the diagnosis. Duffy et al. (2012) describe this patient as a heavy smoker (with a mean consumption of 28 cigarettes per day for 35–40 years) with a high level of nicotine addiction with a history of failed quitting attempts and some smoking-related pathological condition. Even when these patients decide to attempt to stop smoking, the success rate is generally low. For instance, Walker et al. (2006) showed that lung cancer patients start smoking again only 2 months after the surgery, supporting the idea that this population is particularly challenging for smoking cessation specialists (Park et al. 2011).

Since, heavy smoking (smoking more than 20 cigarettes a day, even though different definitions are present in literature; Husten 2009) and smoking related diseases are particularly associated with lung and oral cavity cancer (Gandini et al. 2008), we may expect to find more patients who continue smoking after diagnosis in these pathological conditions. Actually, several studies of non–small-cell lung cancer patients have shown that many patients continue smoking (Gritz 1991; Dresler et al. 1996; Sanderson-Cox et al. 2002) with estimates ranging from 13 to 70 %.

In particular, Walker et al. (2004) in a retrospective study of 49 lung cancer patients reported that patients who were younger and less educated were more likely to return to smoking after surgery. They also found a correlation of relapse with depression and social support. Authors suggested that patients stop smoking immediately before and after surgery, but relapse to smoking remains a problem.

Cooley et al. (2009) in a study on a sample of 94 patients with early stage non-small cells lung cancer reported similar results, but they also found a correlation between smoking and pain. Authors collected data at 1, 2 and 4 months after surgery and their results suggest that the presence of pain is significantly correlated with smoking after surgery.

Schnoll et al. (2005) among 109 lung and head and neck cancer patients, showed that continued smoking after 3 months post-surgery is associated to low quitting motivation and smoking-related beliefs that increase the perception of disadvantages of quitting. In fact, in their study authors found that patients who continue smoking believe that cons of quitting are more than pros. False or biased smoking-related beliefs are frequently present in smokers. Different studies reported that smokers usually show some kind of belief bias, ranging between 20 and 80 % (Masiero et al. 2015). This is also true in oncology settings. Such biased thoughts and beliefs may be exemplified by patients developing the idea that the cancer does not relate to individual choices and behavior but only to uncontrollable factors such as genetic predisposition, environmental pollution, etc. (Arnett 2000).

In particular, smokers tend to underestimate smoking-related risks (Borrelli et al. 2010). For instance, a study of Bock et al. (2001), reported that 47.6 % of smokers admitted to the emergency department for acute respiratory care did not believe that they had a condition that was caused or made worse by smoking.

Furthermore, smokers compared with non-smokers tend to underestimate risks concerning themselves, but they often change their judgment when thinking about others’ risks. This attitude is defined optimistic bias (Weinstein et al. 2005; Weinstein 1989). It drives people to believe that they are protected from the consequences of potential sources of risk and reinforces cigarette smoking. However, some authors (e.g. Milbury et al. 2012; Gritz et al. 2005) have suggested that giving up smoking may be facilitated if patients become aware of the possibility of changing their behavior and in so doing reduce further risks.

Cancer-related emotional distress may favor smoking as well. Indeed, depression is often present in patients, ranging between 3 and 31 % (Krebber et al. 2014). Also blame contribute to exacerbating patients’ psychological distress. Paradoxically, this reaction seems to initiate a vicious circle, which reduces the motivation to start an antismoking program (e.g. Chapple et al. 2004). A study by Shin et al. (2014) on 45 patients and 173 family members who continued to smoke for at least 1 month after the diagnoses reported interesting data about the emotional experience of patients and their relatives. In this study, most smoker patients report to experience guilt toward their family (75.6 %) and to conceal their smoking behavior. Similarly, relatives who continue smoking feel guilt toward patients (63.6 %). Hence, both relatives and patients experience negative emotions and try to prevent conflicts hiding their behavior also to the health personnel.

Long-term abstinence can be hindered by a negative mood, since a correlation between cigarette smoking and depression is well established (McClave et al. 2009). Depressed smokers or smokers with a previous history of major depression generally have a high level of nicotine dependence and a low probability of a successfully attempt to stop smoking.

In oncology settings, depression is a critical issue for patient management. Indeed, cancer patients can experience high levels of worries, fears, anxiety and negative emotions related to the disease and its treatment (surgery, chemotherapy or radiotherapy, periodic clinical meetings, long periods of hospitalization, tests and examinations). Patients may then use cigarette smoking as a coping strategy. Furthermore, the pharmacological properties of nicotine, with its cognitive-enhancing effects, reinforces the perceived valued of cigarettes since they may be used to alleviate treatment-related negative effects on memory and attention.

Spek et al. (2013) found that the decrease of self-efficacy tends to negatively affect smokers’ confidence in their ability to quit smoking. Actually, patients with a high degree of confidence in their power to give up smoking have a higher success rate than smokers with a low confidence. Also, they report less relapses after an attempt to quit (McBride and Ostroff 2003; Elfeddali et al. 2012).

Other context-related variables influence smoking cessation, such as living or working with people who habitually smoke. Indeed, patients who live with smokers are more likely to maintain their habit than are patients who live with non-smokers or in a smoke-free home. Furthermore, social and family support (relatives, friends, colleagues and so on) can be an important factor in promoting smoking cessation even though different studies have shown divergent results (Park et al. 2004). May and West (2000) in a review of the literature found that social support may be beneficial in helping smokers quitting, even if more research is needed to clarify the role of relatives, especially in cancer survivors. However, Schnoll et al. (2002) in a study of 74 patients found that a low readiness to quit smoking after a cancer was strongly associated with the fact of having a relative at home who smoke. After hospitalization, when the patient goes back home, smoking-related cues (e.g. a habitual smoking place; smoking-related daily routine; other people who smoke; sensorial stimuli and the like) may influence their ability to remain abstinent.

This fact has been considered as a serious barrier in helping patients to stop smoking. Indeed, daily exposure to smoking-related stimuli may produce physiological and cognitive changes that favor urgency and craving due to a so-called attentional bias. This cognitive bias is related to the selective properties of the attention. It concerns the attitude of smokers and former smokers to orient their attention to tobacco cigarette smoking cues (Field et al. 2009) favoring the need to smoke. Finally, Ark et al. (1997) found that in head and neck cancer patients heavy alcohol use is a negative predictor of smoking cessation.

### Smoking cessation interventions for cancer patients: state of the art

Smoking interventions may be classified into two main approaches: smoking cessation aids (SCAs) and cognitive behavioral interventions (CBIs). Both methods are used to approach cigarette addiction in healthy adults and in cancer patients and they can be used alone or in combination. However, most research on smoking cessation has been carried out on healthy people, while there are only a few studies available on cancer patients and on survivors (Duffy et al. 2012; see Table 2).

**Table 2:** Clinical trials on smoking cessation from 2000 to 2014

CLINICAL TRIALS 2000–2014								
Year	Authors	Design study	Aim	Sample	Smoking cessation intervention	Follow-up	Disease	Outcome
1993	Gritz et al.	Randomized controlled study	Assessing a provider-delivered smoking cessation counseling program in cancer patients	186	Group 1: usual care including information on the risks of continued smoking and benefits of cessation and physician advice to quit Group 2: usual care advice plus discussion of the person’s readiness to quit, provision of informational booklets, and the physician’s expressions of confidence in the patient’s ability to quit (plus booster sessions during six monthly appointments)	12 months	Head and neck cancers	60 % patients was abstinent (confirmed by cotinine) and among the remaining smokers, consumption dropped by 50 % (12/cigarettes/day). No differences between groups. 39 % of the sample was not assessed at 12 month due to health conditions, death and dropout
1994	Stanislaw, Wewers	Randomized controlled study	To examine the effect of a structured smoking cessation intervention during hospitalization on short-term smoking abstinence	26	Structured counseling smoking cessation treatment during hospitalization followed by five weekly phone calls after discharge	6 weeks (first post discharge visit)	Mix cancer sites	abstinence rates confirmed by cotinine were 75 % for the intervention and 40 % for usual care
1998	Griebel, Wewers, Baker	Prospective, two-group, randomized clinical trial	Assessing the effectiveness of a nurse-managed minimal smoking-cessation intervention among hospitalized patients with cancer	28	During hospitalization, subjects were assigned to a minimal counseling smoking-cessation intervention group (n = 14) or to usual care treatment (n = 14)	6 weeks post intervention	Mix cancer sites	21 % and 14 % of the intervention and usual care group, respectively, were abstinent at 6 weeks
2003	Schnoll et al.	Randomized controlled trial	Assessing physician-based smoking intervention comparing to usual care	432	Control Group (N = 218) Usual care: minimal advice. Intervention Group (N = 217) Intervention: quitting advice; nicotine replacement therapy; self-help smoking cessation guide; telephone number for additional assistance and/or referral to a cessation program; assessed cessation progress during subsequent medical visits	6 months–12 months	Breast, prostate, or testicular cancer or lymphoma	No association found between physician-based smoking intervention and long-term (6 months follow up and 12 months follow up) quit rates
2004	Wakefield et al.	Randomized controlled trial	Evaluating whether motivational interviewing intervention increases quit rates	137	Control Group (N = 63): minimal advice. Intervention Group (N = 74): motivational interviewing intervention (over at 3-months period); nicotine replacement therapy (in patients who smoked more than 15 cigarettes per day), family advice to quit, and an in-person or telephone follow-up conversation. Follow-up at 6-months	6 months	Mixed cancer sites	No evidence to support efficacy of the motivational interviewing intervention
2005	Croghan et al.	Method no stated	Assessing individual counseling intervention comparing to physician advice alone	30	Control Group (N = 11): minimal advice. Intervention Group (N = 19): individual counseling intervention (1 session for 45 min) plus individualized nicotine replacementtherapy	No information available	Lung or Esophageal cancer	No evidence to support efficacy of the individual counseling intervention
2005	Schnoll et al.	Randomized controlled trial	Assessing cognitive-behavioral therapy plus Nicotine ReplacementTherapy (CBT) comparing to General Health Education (GHE)	109	No indication about dimension of control and experimental group. Control group: minimal advice plus transdermal nicotine patches (8 weeks). Intervention group: individual counseling intervention (4 counseling sessions, 1 in-person and 3 over the phone) plus transdermal nicotine patches (8 weeks)	1 month–3 months	Headand neck, lung cancer	No evidence to support efficacy of the tailored CBT intervention both at 1 month and at 3 months follow-up
2010	Thomsen et al.	Randomized controlled multicentre trial	Primary aim: Evaluating the impact of brief smoking cessation interventions on post-surgery complications. Secondary aim: Assessing the efficacy of brief smoking cessation interventions on quit rates	130	Control group (N = 65): minimal advice. Experimental group (N = 65): brief smoking intervention (motivational interviewing plus personalized nicotine replacement therapy)	No information available	Breast cancer	Primary outcome: No observed clinical impact on postoperative complications. Secondary outcome: Brief smoking intervention administered shortly before breast cancer surgery modestly increased preoperative smoking cessation
2010	Schnoll et al.	Double-blind placebo-controlled trial randomized	Assessing the efficacy of bupropion for treating tobacco dependence among cancer patients	246	Control group (N = 132): counseling (5 sessions), transdermal nicotine patches (8 weeks) plus placebo (9 weeks). Experimental group (N = 114): counseling (5 sessions), transdermal nicotine patches (8 weeks) plus Bupropion (9 weeks)	3 months–6 months	Mixed cancer sites	Bupropion has a low impact on quitting rate on patients with depression symptoms, but not in cancer patients without depression symptoms. Bupropion is safe for cancer patients who smoke (in terms of bupropion-related side effects) and may increase QOL for patients with depression symptoms
2011	Park et al.	Pilot study	Assessing the efficacy of a smoking interventions based on behavioral counseling plus varenicline	49	Control group (N = 17): no information available. Experimental group (N = 32): varenicline (12 weeks) plus counseling (9 sessions)	2 weeks–2 months	Thoracic cancer	Varenicline plus counseling increased quitting rate
2012	de Bruin-Visser et al.	Method not stated	Examining the efficacy of nursing-delivered smoking cessation intervention	145	Nursing-delivered smoking cessation program (counseling plus nicotine replacement therapy)	6 months–12 months	Head-and-neck; Lung; Breast; Sarcoma; Bladder cancer	Counseling plus nicotine replacement therapy seem to improve smoking cessation rate both 6 months and 12 months
2013	Bastian et al.	Randomized controlled trial	Assessing the efficacy of proactive telephone counseling combined with a tailored self-directed intervention comparing to tailored self-directed intervention alone	596	Control group (N = 251): transdermal nicotine patches. Experimental group (N = 245): proactive telephone counseling plus transdermal nicotine patches	2 weeks–6 months–12 months	Lung cancer	No difference
2013	Hawari et al.	Prospective observational study	Evaluating the abstinence rates in cancer patients that they underwent to smoking cessation program	210	Tobacco Intervention: medical counselling, pharmacologic management (varenicline or bupropion), plus NRT	3- 6 and 12- months	lung, urinary bladder, head and neck cancers and myeloid leukemia	Abstinence rates declined with time 3 months: relapse related to withdrawal 6 months: relapse rated to personal and professional stressful 12 months: relapse rated to personal and professional stressful
2013	Ostroff et al.	Randomized controlled trial	Assessing the efficacy of short smoking intervention pre-surgical	185	Control group: counseling plus pharmacotherapy. Experimental group: short smoking intervention pre-surgical plus counseling and pharmacotherapy	3 months–6 months	Mixed cancer sites	Short smoking intervention pre-surgical does not increase quit rates both in short term (hospital admission and 3 months) and in long term (6 months)
2016	Price et al.	Randomized controlled trial	Assessing the feasibility, safety, and effect on cessation of varenicline combined with counseling for smoking cessation in patients with cancer	132	Group 1: 12 weeks of varenicline followed by 12 weeks of placebo Group 2: 24 weeks of varenicline All Participants also received behavioral counseling consisting of one 60-min in-person pre-quit counseling visit at week 0 and further in-person counseling sessions at weeks 1, 4, 8, and 12	12 weeks	Mixed cancer cites	40.1 % of patients self-reported to be abstinent at 12 weeks. Abstinence correlated with improvement in cognitive functions

### Smoking cessation aids: SCAs

Smoking cessation aids involve three categories of antismoking strategies: nicotine replacement treatments (NRTs), tobacco free cigarettes—(TFC) and medications (mainly bupropion and varenicline).

Nicotine replacement treatment is very popular among smokers. Generally, a first spontaneous attempt to give up involves NRT (where available without a medical prescription). They provide nicotine, stabilizing its level in the blood in order to avoid a withdrawal syndrome after having stopped smoking. They are available in different forms including transdermal nicotine replacement products, patch, gum, lozenge, inhaler, spray and sublingual tablets. Commonly, the treatment lasts for 10–12 weeks, but it is often prolonged to 6 months. The type of NRTs chosen depends on personal preference, but clinical considerations should be issued too.

Two NRT groups, depending on the administration method, are usually described. The first group includes NRTs that require daily administration, generally based on the use of patches. The second group include protocols based on multiple administrations a day, so-called short-acting NRT (Nides 2008).

The main aims of NRT are three: craving reduction, withdrawal control and abstinence promotion (Stead et al. 2008). A growing body of scientific literature stresses that NRT helps patients to give up smoking (Fiore et al. 2000). Pharmacological trials in particular have shown that the combination of multiple forms of NRT improves the interruption success rate (Ferguson et al. 2011) also in medical settings (Hurt et al. 2009). Also, NRTs have few side effects, being the most prevalent a transient nausea (Fiore et al. 2008). Their principal weak point is the low compliance, since after a short period many people interrupt the smoking cessation program, believing that they are able to control the situation without further help (Balmford et al. 2011). NRT may be successfully used with cancer patients, generally in association with some form of counseling and/or other pharmacological treatment (see Table 2). For instance, a retrospective study by Garces et al. (2004) showed that a combination of NRT and other behavioral and pharmacological treatment is as effective in head and neck cancer patients as in the general population.

A particular type of NRT involves tobacco-free cigarettes (TFCs) also called e-cigarettes. These devices have rapidly become widespread over the last 5 years, and are often used by smokers to reduce the daily number of cigarettes, or to smoke in places where tobacco cigarettes are not allowed.

A TFC device is composed of a battery-powered device that produces a vapour for inhalation. The steam contains humectants, flavours, and pure nicotine. Liquids used in e-cigarettes do not contain tobacco and do not require combustion. They may or may not contain nicotine.

TFCs containing nicotine can be considered a NRT, albeit with specific and distinct characteristics. Firstly, comparing TFCs with traditional NRTs, the former deliver a modest amount of nicotine (Fagerström and Eissenberg 2012). Secondly, TFCs present a better usability and they are more appealing for smokers since they can be used when needed by a well-known modality, i.e. taking a cigarette-like device to the mouth and inhaling. In doing so, smokers not only may react to an immediate need or desire, but they can enact a gesture associated with their previous status (cigarette smokers), thus eliciting positive emotions and satisfying psychological expectations. Thirdly, TFCs may be used ad libitum enabling patients to feel free to control their behavior instead of feeling an under external imposition. For these reasons, it has been suggested that TFCs fit in very well with both the psychological and physiological needs of smokers who want and/or need to stop.

Some clinical trials revealed that e-cigarettes attenuate the craving for cigarettes (Wagener et al. 2012; Bullen et al. 2013). Furthermore, a recent review of the field showed that TFCs may be considered safe, with few adverse events and limited toxicity (Farsalinos and Polosa 2014). In the general population, nicotine TFCs has been showed to be more efficacious in reducing withdrawal symptoms than nicotine-free TFCs (Etter and Bullen 2011). However, also the latter may be considered valid options in oncology settings when supported by counselling, as shown by ongoing studies (Cipolla 2012; Lucchiari et al. 2016). The use of nicotine-free TFCs allow drug-free smoking cessation treatments, and this fact could be particularly interesting for patients undergoing aggressive therapies or at high risk for cardiovascular events. Though some others studies did not find and effect of the use of TFCs in a population of cancer patients (e.g. Borderud et al. 2014; Barton 2015), we argue that only long run longitudinal studies on targeted population and using randomized design will clarify the role of TFCs devices to help patients to quit smoking.

At the time of publishing, there is a clear gap in the knowledge about the feasibility, efficacy and safety of TFCs in oncology settings. In consideration of the lack of evidence, it is not possible to recommend a systematic use of TFCs with cancer patients (Cummings et al. 2014). However, a number of studies are now open on over the world. At our institution, two trials are currently ongoing to analyse how TFCs with and without nicotine might be included in clinical protocols both during cancer treatments and in early diagnosis screening programs.

Lastly, the pharmacological approach to smoking cessation is mainly based on two medications: bupropion and varenicline, both of which are extensively used in clinical practice. Bupropion is an atypical antidepressant that promotes cessation probably by reducing the stimulating effect of neurotransmitters, being an inhibitor of dopamine and norepinephrine reuptake. It counteracts craving and smoke-related withdrawal symptoms (Jorenby et al. 2006). In addition, it counters emotional distress during abstinence, which is a very important characteristic to consider for cancer patients. Indeed, as noted above, cancer patients frequently experience high levels of depression, and smoking interruption can drastically increase this because of psychological and physiological cigarette dependence.

Bupropion has been administered to cancer patients during treatment, and it was found to improve their mood, facilitating the smoking cessation success. In particular, Schnoll et al. (2010) designed a double-blind, placebo-controlled trial ran on a sample of 246 cancer patients (mix cancer sites). In the first arm of the study, patients received 5 counseling session, 8 weeks of NRT and 9 weeks of bupropion. In the second arm, patients received the same treatment of group 1, but bupropion was substituted by a placebo. While NRT and counseling was found to be effective in non-depressed patients, bupropion showed to be beneficial for those patient who reported depressive symptoms. Benefits were found both for mood and for smoking cessation rate.

Bupropion has other relevant properties, e.g. it reduces post-cessation weight gain (Martinez et al. 2009), and it can be used even with patients with cardiovascular problems. However, the use of bupropion should be avoided for patients receiving active treatments for brain or other central nervous system cancers, and for those patients with a history of seizures, bulimia or anorexia nervosa. Furthermore, the use of this agent must be carefully weighed when dealing those patients receiving chemotherapy with direct CNS effects due to the increased potential for seizure. More research in the safety of bupropion during cancer therapy is warranted, even though no further contraindications are suggested (Klosky et al. 2007).

A further pharmacological treatment for smoking dependence involves the use of varenicline. This is a partial agonist of nicotinic acetylcholine receptor subtype α_4_β_2_, reducing dopamine stimulation. A study by Gonzales et al. (2006) reported that a 12-weeks varenicline-based treatment counters craving, urgency and withdrawal symptoms in healthy smokers. If properly administered, varenicline causes cigarettes to lose their habitual pleasure, favouring the extinction of the smoking habit (Baraldo et al. 2012; Ebbert et al. 2010). This is particularly true for smokers with a low motivation to give up and/or with important nicotine withdrawal symptoms (Nides 2008). In the general population, varenicline is considered the best option to increase the probability of success of a smoking cessation program, reaching a 25–30 % of abstinences within the first year (Issa et al. 2013; Mills et al. 2012). However, a study by Hawari et al. on a heterogeneous sample (n = 199) of cancer patients (mix cancer sites) failed to find any significant advantage by the use of varenecline in association with NRT at 3 months with respect to NRT alone.

Nausea, abnormal dreams, upper respiratory tract infection and insomnia are the most commonly reported adverse events, and these are generally mild and self-limiting. Although it has been associated with depression, suicidal behaviour and myocardial infarction (depression is actually a relative contra-indication; Brophy 2011), a recent meta-analysis showed that there is no clear evidence that these side-effects are causally related to the drug use (Prochaska and Hilton 2012). Varenicline should be avoided with pregnant, nursing, in patients with renal impairment or patients younger than 18, but generally appears safe in treating tobacco dependence of patients with cancer. Furthermore, the use of varenicline seem to support cognitive functions (Clinical Practice Guidelines in Oncology: Smoking Cessation 2015). In particular, a recent study by Price et al. (2016) on a sample of 132 smokers with a diagnosis of cancer found that varenicline was well tolerated by patients. They designed a two arms randomized study. Patients received or 12 weeks of varenicline followed by 12 weeks of placebo or a full 24 weeks of varenicline. However, at the moment of publication only data about the first 12 weeks of varenicline are available. The pharmacological treatment was combined with five standardized brief smoking cessation counselling sessions. About 40.1 % of smokers stopped after 12 weeks of treatment, a datum comparable with results found in the general population. Side effects symptoms were present (in particular, nausea and sleep problems) but they did not prevent patients to complete the treatment. Furthermore, abstainers at 12 week showed a significant decrease in cognitive deficits. The authors did not report data about longer-term assessment points and the placebo condition. Furthermore, the cancer stage is very heterogeneous (in the 33.3 % is not specified) and many patients (25.8 %) are in remission. Under this condition is difficult to evaluate the real ability of patient to tolerate the treatment side effects.

The use of vareniclin in oncological setting require more research in order to establish in which context it should be beneficial.

### Cognitive behavioral interventions (CBIs)

Cognitive behavioral interventions (CBIs) are based on a psychological approach to smoking aimed at supporting people in changing their behavior by a cognitive restructuring. Low-intensity or high-intensity interventions may be used. The low-intensity interventions are more frequently used in oncology settings, in screening programs as well as in other clinical settings. They are characterized by self-help tools and the use of so-called minimal advice. Normally, the self-help tools are booklets, brochures, and informative paper-based support materials, although video clips and computer-based tools are also now available. These tools convey general information about the consequences of smoking and the benefits of giving up.

The main advantage of self-help tools is that they target a large population; unfortunately, their effects are generally poor (Lancaster and Stead 2005).

The minimal advice is a short list of recommendations, requiring just some minutes, focused on why it is important to consider to stop smoking and how (and where) it is possible to address the issue, e.g. starting an antismoking program. This approach is particularly useful in a clinical setting, where trust between patients and physicians is already established and where external but affordable resources are available (e.g. an antismoking center).

When meeting a patient, oncologists and/or nurses may merely explain the importance of interruption of smoking in cancer care and the impact on treatment, but they do not directly suggest strategies or therapies (Emmons et al. 2013).

The minimal advice consists of the evaluation of the personal smoking history and a few suggestions. Several studies reported a delay of specific interventions for patients who smoke after the diagnosis, probably due to a “beating the disease” priority. The urgency of the disease focuses the doctor’s attention on the assessment and the cure of clinical conditions, while other aspects may go untreated.

The high intensity interventions involves a counseling approach. Behavioral counseling has been defined as a relationship between an expert and a client, focused on behavior to be changed. Normally, each session lasts 15–30 min.

A broadly used tool is the motivational interview aimed to understand the subjects position within the so-called cognitive spiral of change (Prochaska and DiClemente 1983), and to activate motivational drivers. Previous research has stressed that a high motivational level can facilitate smoking cessation and abstinence, while a low motivational level negatively affects the success rate. The motivational level is considered as a benchmark to decide which strategy to opt for.

Behavioral counseling can be used in oncology settings and continued during the follow-up scheduling. Generally speaking, behavioral counseling starts during the hospitalization and should be continued after discharge. It has been shown that it increases the giving-up rate after 6 and 12 months (Rigotti et al. 2008) in hospitalized patiens. Another widespread model, very useful in hospital settings, is telephone counseling. Habitually, it is used in combination with traditional (on-site) counseling (Stead et al. 2006). This intervention can be proactive or reactive (Lichtenstein et al. 1996). The proactive approach targets patients already prompted to give up, and then may be used to monitor the ongoing situation over time. Instead, the reactive approach is based on specific needs of patients (Stead et al. 2008). A review on telephone counselling has stressed that it sustains abstinence in the long run (Tzelepis et al. 2011). The smoking cessation counseling should be accompanied by a set of recommendations regarding the need of a generalized lifestyle change (Anderson et al. 2002). This aspect of counseling could be very important in oncology setting, since physical activity may have a positive impact on quality of life and wellbeing (Gill et al. 2013), thus potentially acting as the trigger of a virtuous circle. Health personnel could use their privileged role to encourage changes in this direction and redirect patients to external services after discharge. Generally, speaking counseling may be considered effective in supporting smoking attempts in oncology settings in association with smoking cessation aids (Klemp et al. 2016). For instance, Park et al. (2011) studied the effect of antismoking counselling in association with a pharmacological tobacco treatment (Varenicline) in a sample of 49 cancer patients with a thoracic cancer, showing the positive impact of this combined treatment. In this study, the intervention consisted in a 12-week program (Varenicline and counseling). Patients in the treatment group received a median of 9 counseling sessions structured according the 5 As brief counseling model (Ask, Advise, Assess, Assist, Arrange follow up). Authors report that the prevalence abstinence rate at 12 week follow-up was 40.6 in the intervention group and 14.3 in the control group.

Similar results were obtained by de Bruin-Visser et al. (2012) using a 1-year minimal intervention counseling program delivered by nurses (one visit a week in the first month and one every other month with the endpoint at 12 months). Authors included in their study 145 patients with mix cancer sites and found a positive effect of the counseling, since at 6 months the 40 % of patients had stopped smoking, and 33 % was abstinent at 12 months. During this period, most patients also use some kind of nicotine substitutes (83 %), but authors report no association between abstinence and the use of nicotine replacement products. However, Bastian et al. (2013) failed to find any positive relationships between a telephone-based counseling program and smoking cessation in a large sample of 596 lung cancer patients. In particular, authors compared an at-distance counseling program (6 calls) based on motivational interviewing techniques with tailored self-directed materials (n  =  245) and a minimal intervention based only on tailored self-directed materials (n  =  251). In both groups nicotine patches was al used if not contraindicated. Their results showed no differences between the two groups, even though in younger patients (<50) the abstinence rate was higher (16 vs. 4 %) at 2 weeks post-surgery. This study underline once more that lung cancer patients need intensive interventions to stop smoking.

Other studies have tested the efficacy of a strategy based on a brief physician intervention, showing that low intensity intervention have poor power in helping patients quitting attempts. In particular, Schnoll et al. (2003) compared two large groups of patients (mix cancer sites). The intervention group (n = 217) received a minimal advise and information about the benefits of quitting and smoking cessation treatment. Furthermore, patients in this group received a self-help smoking cessation guide a telephone line for additional assistance or information. The control group (n = 218) received only a minimal advise about smoking by their physician. At 6 months, 14.4 % patients in group 1 and 11.9 % in group 2 had stopped smoking, showing no significant differences between the two arms of the study.

Though the studies we have considered did not directly analyzed the cost-effectiveness of smoking cessation treatment in oncology settings, de Bruin-Visser et al. (2012) suggest to estimate cost-effective analysis for cancer patients using by studies in the general population. In particular, Song et al. (2002) found that NRT for smoking cessation is cost-effective. The results of the decision analyses indicated that, as compared with advice or counseling alone, the incremental cost per life-years saved is about $1441–$3455 for NRT, $920–$2150 for bupropion, and $1282–$2836 for NRT plus bupropion. The cost-effectiveness of adding NRT and bupropion to advice or counseling for smoking cessation is better than many other accepted health care interventions. Hoogendoorn et al. (2010) analyzed the long-term cost-effectiveness in patients with chronic obstructive pulmonary. They reported that pharmacological treatments in combination with intensive counselling was cost saving compared with intensive counselling alone, and that the latter in turn was more effective than minimal intervention.

Finally, Meenan et al. (1998) analyzed the cost-effectiveness of a hospital-based smoking cessation intervention reporting that the cost of the research intervention was $159 per smoker, and incremental cost per incremental quit was $3697. Incremental cost per incremental discounted life-year saved ranged between $1691 and $7444, much less than most other routine medical procedures.

## Conclusions

A cancer diagnosis may represent a fundamental teachable moment for smokers, leading them to give up smoking or to significantly reduce daily cigarettes. Most former smokers declare that they decided to quit due to physical issues or the fear of future health consequences of smoking (Gallus et al. 2013). Furthermore, a regression analysis on 15,489 smokers participating in lung cancer screening studies reviled that smoking cessation is strongly associated with screening abnormalities and that efficacious smoking cessation programs should be integrated within screenings (Tammemägi et al. 2014). Despite this evidence, few studies have directly addressed the problem of how to help a patient with cancer to abandon unhealthy habits. Additionally, even though some recommendations exist, no specific and validated protocols are recognized as a valid and shared antismoking approach in oncology settings. As a consequence of this attitude a cancer patient is rarely concretely and systematically supported in a smoking cessation journey (e.g. Nayan et al. 2011; de Bruin-Visser et al. 2012), with the result that though many patients developed a good motivation to stop, few actually remain abstinent in a long run. Thus, the emotional trigger of the diagnosis and the motivational context of the clinical setting succeed only in helping patients to suspend cigarette smoking for a short period (e.g. Land et al. 2011) but not as a sound lifestyle change.

From a psychosocial point of view, the fact that motivation to stop level regresses to its pre-diagnostic level is an expected phenomenon (Slovic et al. 2004). Indeed, emotions have a time-limited effect and their impact on decision-making processes (e.g. whether to attempt quitting or not) rapidly fades after an emotional burden. Emotions must be considered as short-time cognitive modulators, being able to focus attention on risk-related behaviors and the need to avoid them in a specific spatial and temporal context (Pravettoni et al. 2012). Consequently, time matters, since the more time pass between diagnosis and treatments the more the motivation to quit decreases.

Furthermore, in order to help oncology patients to reduce smoking-related risks, it is vital to know the general characteristics of the context in which the emotions arose. If a patient learns in a specific, clinical setting (a surgery and/or oncology department) that smoking is dangerous and that quitting could be a good solution for their future health, this setting will be associated with that emotionally-laden knowledge. Consequently, patients will develop a situated learning that will motivate their choices. However, when a patient goes back home the context will dramatically change, activating previous learning and habits, e.g. smoking while watching TV or after dinner. Here the motivation to quit will rapidly vanish if not adequately supported by prior interventions and adequately sustained by a continuous help, e.g.by a telephone counseling. Actually, in a study on 721 lung cancer survivors, smoking cessation success was found to be negatively associated with smoking exposure at home (Eng et al. 2014).

Consequently, we argue that in order to help patients to give up smoking, the health personnel working in oncology settings should be prepared to offer a complete program aimed at promoting health by integrating new perspectives and attitudes in patients’ values system. Cancer centers should promote smoking cessation applying guidelines and doing more research on smokers’ needs in order to implement tailored and effective interventions (Mazza et al. 2010; Cataldo et al. 2010).

In particular, even if it is very important to consider the cancer treatment a teachable moment to help a concrete lifestyle change, we claim that emotions should not be used as levers to force their change, but only as initial triggers. Indeed a social-cognitive approach to patients suggests that physicians and nurses should use their position within the clinical setting to drive patients through a sound learning process, instead of causing just a time-limited suspension of a dysfunctional behavior. In particular, a cognitive perspective should be used in order to understand which personal beliefs sustain the smoking behavior, since biased evaluations could prevent patients success in their attempts to quit (Masiero et al. 2015; Pepper et al. 2014). However, to achieve this aim the hospital personnel should be educated by specific training and motivated to adopt the best practices by hospital initiatives.

Overcoming the limits of the classical minimal advice approach, which could be just the initial part of a more complex process, the social-cognitive intervention should be:

- *Integrated* the smoking cessation treatment should be experienced as an integral part of the cancer management. In particular, nurses could serve this aim by introducing the issues of smoking and smoking cessation and by evaluating the tobacco dependence and the motivation to stop so to address patients to dedicated services.
- *Personalized* the therapeutic options provided to patients should be compatible with their values, needs and attitudes. Furthermore, it should address a whole lifestyle change.
- *Proactive* offering patients continuity in treatments, avoiding offering merely on-demand support or suggestions.
- *Reactive* providing patients dedicated treatments or support when needed, independently of any therapeutic scheme.

This approach requires a multidisciplinary effort since physicians, nurses and behavior change specialist should work together each having specific roles (Lucchiari et al. 2013). In particular, physicians should introduce the issue of smoking within the general treatment, using the therapeutic alliance to gain the attention of the patient and guide the subsequent cognitive assessment of smoking-related risks and benefits of quitting. Some studies (e.g. Mazza et al. 2010) have reported the fear of physicians to suggest antismoking programs due to the fragile emotional situation of patients, and the potential to cause distress responses. Nonetheless, an antismoking pathway should not be experienced by patients as a further stressor, and we suggest that health personnel could play a vital role in helping patients to approach the problem in a balanced fashion, to reduce stress and maximize benefits. Since a number of studies on cancer patients demonstrate moderate smoking cessation success following the general principles of evidence-based smoking cessation protocol (Gritz et al. 2014) a targeted and interdisciplinary approach should be adopted using systematic monitoring of patients’ needs and behavior in order to sustain long-term abstinence (Hawari et al. 2013). However, when this is not possible, physicians and nurses should act as valuable information carriers, addressing relevant issues and encouraging patients to search for support in external services.

Health personnel should provide detailed information about smoking hazards (both before, during and after cancer) and support patients’ motivation to start an attempt to quit (Zwar et al. 2011). This support should be based on a social-cognitive perspective, since any information and/or advice provided should be aimed at breaking down false beliefs and cognitive biases and at reinforcing the emotional, physical and social benefits of the change. Consequently, personnel should be well trained in performing this task and they should be aware of the important role they are playing in the whole process. This mean that physicians and nurses are regarded not only as information bearers, but also as change facilitators, potentially making a great difference in the result (Cooley et al. 2008). Finally, specialized counselors should follow the entire clinical pathway by regular meeting (potentially also at a distance, e.g. by telephone) to provide emotional support, sustain motivation, and approach specific concerns. This should also include behavioral interventions (e.g. helping patients to manage anxiety states, sleep disturbances and the like).

Physicians, nurses and specialists must therefore work in an integrated fashion, able to exchange information, keep track of the patients’ history and adequately respond to patients’ demands throughout all the oncology treatment. This also means that when patients need help, e.g. in the use of antismoking medications or in following specific procedures, they can easily find practical support, as some studies have shown that patients often fail to continue the antismoking treatment due to practical problems (e.g. McBride and Ostroff 2003). Indeed, a whole and integrated approach will get patients into a protection context, where emotional, behavioral and practical issues are continually monitored and supported. However, the future success of tobacco smoking programs in oncology settings will depend on the level of knowledge education and motivation of health personnel.
